# Increased native T1-values at the interventricular insertion regions in precapillary pulmonary hypertension

**DOI:** 10.1007/s10554-015-0787-7

**Published:** 2015-10-16

**Authors:** Onno A. Spruijt, Loek Vissers, Harm-Jan Bogaard, Mark B. M. Hofman, Anton Vonk-Noordegraaf, J. Tim Marcus

**Affiliations:** Department of Pulmonary Medicine, VU University Medical Center, Amsterdam, The Netherlands; Department of Physics and Medical Technology, ICaR-VU, VU University Medical Center, de Boelelaan 1117, PK-1Y138, 1081HV Amsterdam, The Netherlands

**Keywords:** T1-mapping, Myocardium, Non-contrast T1, Pulmonary hypertension

## Abstract

Cardiac magnetic resonance imaging of the pressure overloaded right ventricle (RV) of precapillary pulmonary hypertension (PH) patients, exhibits late gadolinium enhancement at the interventricular insertion regions, a phenomenon which has been linked to focal fibrosis. Native T1-mapping is an alternative technique to characterize myocardium and has the advantage of not requiring the use of contrast agents. The aim of this study was to characterize the myocardium of idiopathic pulmonary arterial hypertension (IPAH), systemic scleroderma related PH (PAH-Ssc) and chronic thromboembolic PH (CTEPH) patients using native T1-mapping and to see whether native T1-values were related to disease severity. Furthermore, we compared native T1-values between the different precapillary PH categories. Native T1-mapping was performed in 46 IPAH, 14 PAH-SSc and 10 CTEPH patients and 10 control subjects. Native T1-values were assessed using regions of interest at the RV and LV free wall, interventricular septum and interventricular insertion regions. In PH patients, native T1-values of the interventricular insertion regions were significantly higher than the native T1-values of the RV free wall, LV free wall and interventricular septum. Native T1-values at the insertion regions were significantly related to disease severity. Native T1-values were not different between IPAH, PAH-Ssc and CTEPH patients. Native T1-values of the interventricular insertion regions are significantly increased in precapillary PH and are related to disease severity. Native T1-mapping can be developed as an alternative technique for the characterization of the interventricular insertion regions and has the advantage of not requiring the use of contrast agents.

## Introduction

Precapillary pulmonary hypertension (PH) is characterized by an increase in pulmonary vascular resistance (PVR) and right ventricular (RV) adaption to the increased load is one of the main determinants of patient outcome [1]. A well-established method to non-invasively characterize the myocardium is the assessment of late gadolinium enhancement (LGE) by cardiac magnetic resonance imaging (CMRI). Previous studies in PH patients showed delayed enhancement of the interventricular insertion regions [2–5] and linked this phenomenon to focal fibrosis [4, 6]. In addition, LGE was shown to correlate with disease severity [2, 4, 5]. A major drawback of the assessment of LGE is the need for the administration of the contrast agent gadolinium. Its toxicity due to depositions in other parts of the body, for example in the brain [7], is not completely understood, but gadolinium administration is contra-indicated in patients with renal insufficiency. Moreover, the LGE technique is not suitable to detect more diffuse myocardial pathologies since the calculation of the threshold of abnormal enhancement depends on a reference area of myocardium [8].

An alternative technique to characterize the myocardium is native T1-mapping. T1-mapping quantifies the T1 relaxation time per pixel tissue and different tissues show a characteristic range of T1-values [9–11]. Native T1-values increase when the heart is affected by edema and diffuse or focal fibrosis [12–20]. To quantify native T1-values, there is no need for a reference area of myocardium, making it possible to directly quantify the total myocardium. Moreover, native T1-values can be determined without the use of contrast agents [10].

Therefore, the aim of this study was to characterize the myocardium of idiopathic pulmonary arterial hypertension (IPAH), systemic scleroderma related PH (PAH-Ssc) and chronic thromboembolic PH (CTEPH) patients using native T1-mapping and to see whether native T1-values were related to disease severity. Furthermore, since the difference in presence of histologically confirmed myocardial fibrosis between these forms of precapillary PH vary between studies [21–23], we compared native T1-values between the different precapillary PH categories.

## Methods

### Subjects

We retrospectively analyzed all available native T1-mapping data of IPAH patients (n = 46), PAH-SSc patients (n = 14) and CTEPH patients (n = 10) scanned between June 2011 and March 2014 in the VU University Medical Center. Data was acquired in the context of an ongoing prospective research program to investigate the role of CMRI in the evaluation of PH patients. The study was approved by The Medical Ethics Review Committee of the VU University Medical Center. Since this study did not fall within the scope of the Medical Research Involving Human Subjects Act (WMO), the study was approved without requirement of an informed consent statement. IPAH, PAH-SSc and CTEPH were diagnosed according to ATS/ERS guidelines [24]. Both treatment naïve patients and patients under optimal PAH-therapies were included in the study. Patients with left sided heart failure and congenital heart disease were excluded from this study. Furthermore, native T1-mapping was performed in ten healthy volunteers, who gave written informed consent for usage of the data for this study.

### CMRI protocol

Native T1-mapping was acquired on a Siemens 1.5 T Avanto scanner. A single breath-hold Modified Look-Locker Inversion-recovery (MOLLI) pulse sequence was used on a mid-ventricular short axis imaging plane. Three, three, and five non-segmented images were acquired at end-diastole within 17 heart beats to sample the recovery of longitudinal magnetization after the inversion pulse. Minimal inversion time was 100 ms [25]. Inplane motion correction was applied. Motion correction was applied by exploiting the known exponential form of inversion recovery and treating the motion and inversion recovery as a joint estimation problem. This was performed with the generation of a series of motion free synthetic inversion recovery images which were used at each inversion time for registration with the measured MOLLI images [26].

### CMRI analyses

**Fig. 1:**
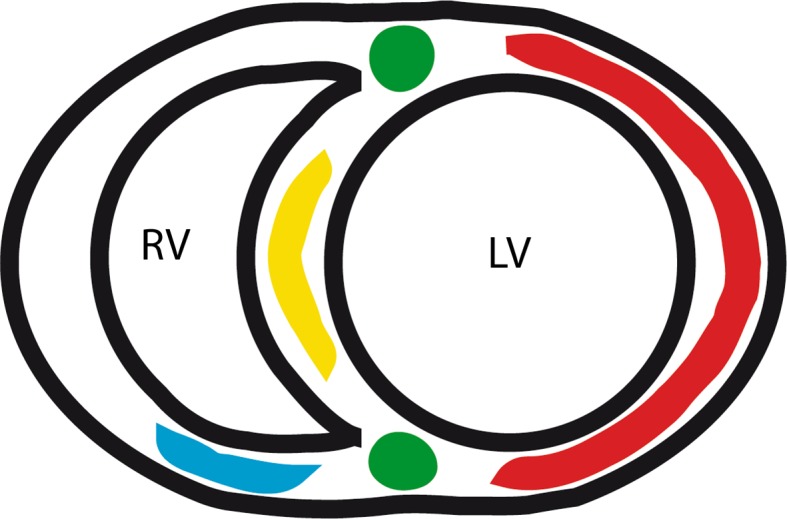
Schematic illustration of ROIs. Schematic illustration of a mid-ventricular short-axis image. ROIs of the different regions are marked with *colors*. ROIs of the LV free wall (*red*) covered the total LV free wall. ROIs of the RV free wall (*blue*), were only analyzed in the inferior part of the RV free wall. Attempts to cover the total RV free wall failed because in a majority of the patients the free wall was too thin, resulting in unreliable native T1-values due to partial volume effects. ROIs of the interventricular septum are marked in *yellow*. ROIs of the interventricular insertion regions are marked in *green*

Native T1-values were assessed using regions of interest (ROIs) at the interventricular insertion regions, the RV free wall, LV free wall, interventricular septum and interventricular insertion regions on mid-ventricular short axis T1-maps. ROIs were manually drawn as illustrated in Fig. 1. ROIs were carefully assessed and the borders of the myocardium were avoided to prevent partial volume effects due to surrounding tissue or the blood pool. ROIs in the RV free wall could be accurately positioned in all patients in the inferior part of the RV free wall (Fig. 1). We attempted to draw ROIs of the total RV free wall, but this was not feasible in the majority of patients because the RV free wall was too thin. Native T1-values of the RV wall could not be assessed in the control subjects because in all control subjects the RV wall was too thin. T1-values of the interventricular insertion regions were averaged over the inferior and superior insertion regions. Ventricular volumes were assessed as described before [1].

### Statistical methods

Data are presented as mean ± standard deviation, unless stated differently. In PH patients, native T1-values between the RV free wall, LV free wall, interventricular septum and interventricular insertion regions were compared using repeated measures one-way ANOVA with Bonferroni post-hoc correction. In control subjects, native T1-values between the LV free wall, interventricular septum and interventricular insertion regions were compared using repeated measures one-way ANOVA with Bonferroni post-hoc correction.

Native T1-values of the different regions of the myocardium between IPAH, PAH-SSc and CTEPH patients and healthy controls were compared using one-way ANOVA with Bonferroni post-hoc correction.

In PH patients, Pearson correlation analysis were applied to assess the relation between native T1-values and hemodynamic and cardiac parameters of disease severity: right atrial pressure (RAP), mean pulmonary artery pressure (mPAP), pulmonary vascular resistance (PVR), cardiac index (CI), RV stroke volume index (SVI), RV end-diastolic volume index (RVEDVI), RV end-systolic volume index (RVESVI), RV ejection fraction (RVEF) and NT Pro-BNP.

All analyses were performed using SPSS for Windows version 20.0 and Graphpad Prism for Windows version 5.0. *p* values <0.05 were considered statistically significant.

## Results

Patient characteristics, hemodynamics and standard CMRI measurements are summarized in Table 1. Control subjects were significantly younger than PH patients (20 ± 1 and 54 ± 16 *p* < 0.001). 40% of the control subjects were female. Heart rate (HR) was not significantly different between control subjects (74 ± 11 bpm) and PH patients (79 ± 14 bpm).

**Table 1:** Patient characteristics

	N = 70 (IPAH n = 46; PAH-SSc n = 14; CTEPH n = 10)	Control subjects (n = 10)	*p* value
Female (%)	73	40	0.04
Age (years)	54 ± 16	20 ± 1	<0.001
Heart rate (bpm)	79 ± 14	74 ± 11	0.280
Hemodynamics			
mPAP (mmHg)	47 ± 13	–	
PAWP (mmHg)	8 ± 3	–	
PVR (dyn s/cm^5^)	634 ± 342	–	
RAP (mmHg)	7 ± 4	–	
CI (L/min/m^2^)	3 ± 1	–	
CMRI			
RVEDVI (mL/m^2^)	82 ± 38	–	
RVESVI (mL/m^2^)	51 ± 37	–	
RVSVI (mL/m^2^)	30 ± 12	–	
RVEF (%)	42 ± 16	–	
LVEF (%)	65 ± 11	–	
NT ProBNP (ng/L)	1393 ± 2283	–	

*mPAP* mean pulmonary artery pressure, *PAWP* pulmonary arterial wedge pressure, *PVR* pulmonary vascular resistance, *RAP* right atrial pressure, *CI* cardiac index, *CMRI* cardiac magnetic resonance imaging, *RVEDVI* indexed right ventricular end-diastolic volume, *RVESVI* indexed right ventricular end-systolic volume, *RVSVI* right ventricular stroke volume index, *RVEF* right ventricular ejection fraction, *LVEF* left ventricular ejection fraction

### Native T1-values in PH patients and control subjects

**Fig. 2:**
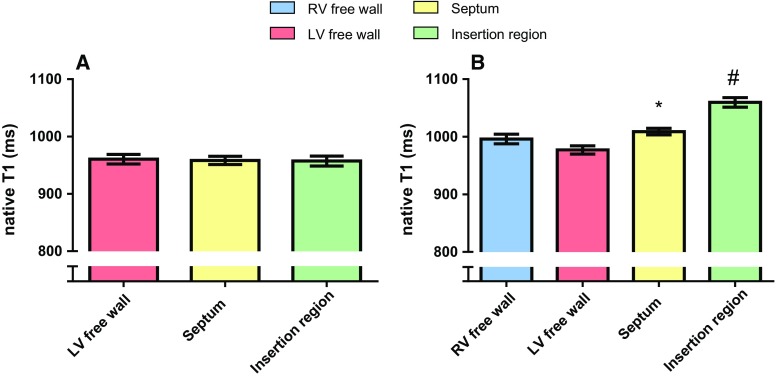
Native T1-values (ms) of the RV free wall, LV free wall, interventricular septum and the interventricular insertion regions. Data is presented as mean and standard error of the mean. **a** Regional differences in the myocardium of control subjects. No regional differences were found in control subjects. **b** Regional differences in the myocardium of PH patients. T1-values of the RV free wall of PH patients were not different from T1-values of the LV free wall and interventricular septum. *T1-values of the interventricular septum of PH patients were significantly higher compared to native T1-values of the LV free wall. ^#^Native T1-values of the interventricular insertion regions of PH patients were significantly higher compared to native T1-values of the RV free wall, LV free wall and interventricular septum

**Fig. 3:**
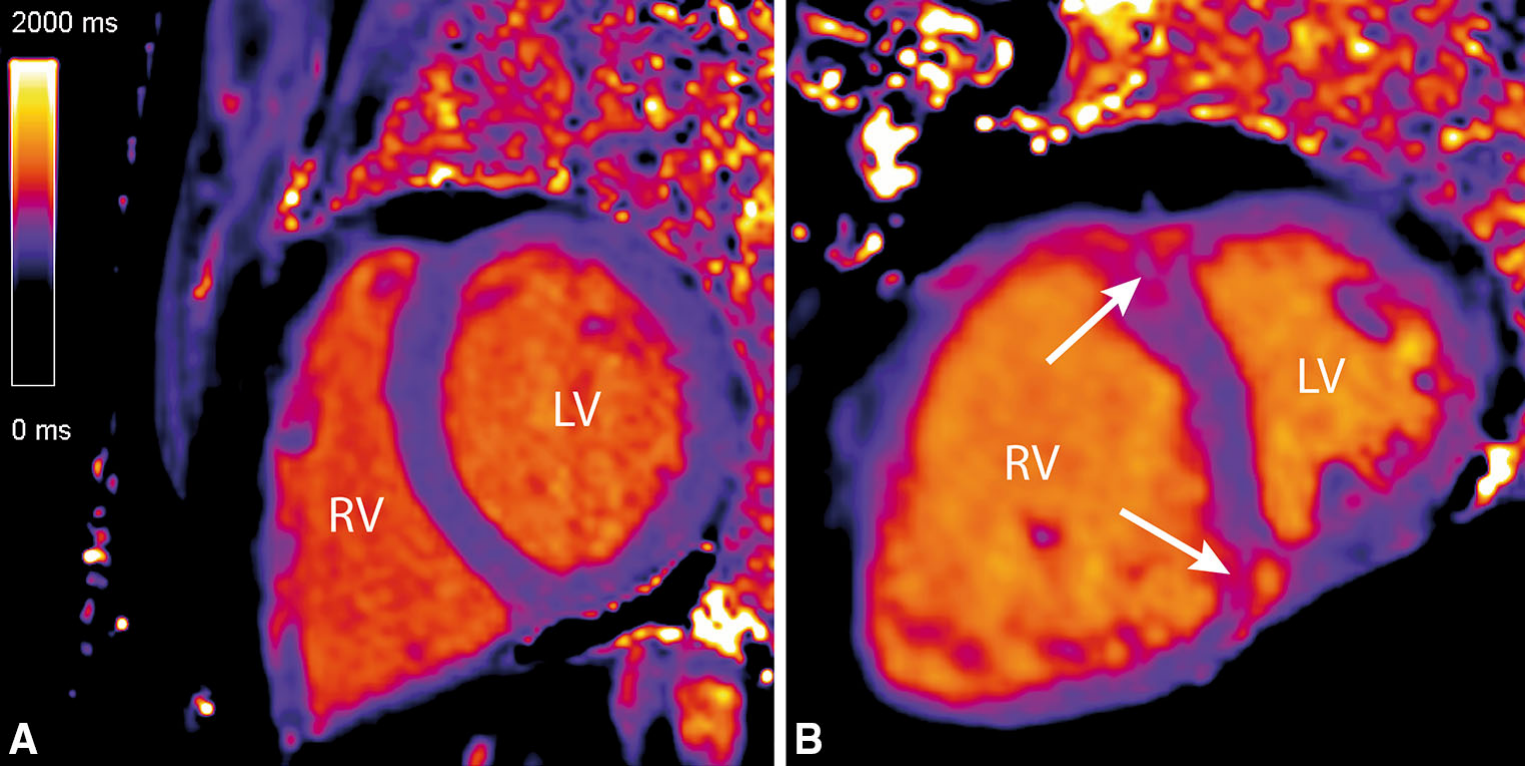
Native T1-maps showing increased native T1-values at the interventricular insertion regions. Two examples of native T1-maps of a control subject (**a**) and an IPAH (**b**) patient. The *white arrows* indicate the increased native T1-values at the interventricular insertion regions

In PH patients, native T1-values of the interventricular insertion regions (1060 ± 70 ms) were significantly higher than the native T1-values of the RV free wall, LV free wall and interventricular septum (Figs. 2, 3). Native T1-values of the RV free wall (996 ± 69 ms) were not significantly different from the native T1-values of the LV free wall (977 ± 60 ms) and interventricular septum (1009 ± 48 ms). Native T1-values of the LV free wall were significantly lower than the native T1-values of the interventricular septum.

In control subjects, no differences were found between native T1-values of the LV free wall (961 ± 26 ms), interventricular septum (958 ± 23 ms) and interventricular insertion regions (957 ± 27 ms) (Fig. 2).

### Relation between native T1-values of the interventricular insertion regions and disease severity in PH patients

**Fig. 4:**
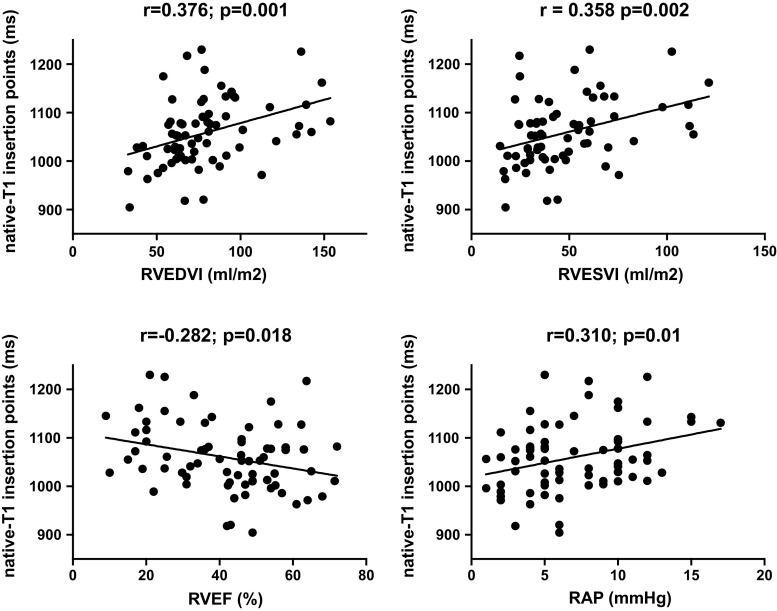
Correlations between native T1-values of the interventricular insertion regions and RVEDVI, RVESVI, RVEF and RAP in PH patients. Native T1-values of the interventricular insertion regions of PH patients were significantly related to RVEDVI, RVESVI, RVEF and RAP. *RVEDVI* right ventricular end-diastolic volume index, *RVESVI* right ventricular end-systolic volume index, *RVEF* right ventricular ejection fraction, *RAP* right atrial pressure

In PH patients, native T1-values of the interventricular insertion regions were significantly related to RAP (Pearson r = 0.310; *p* = 0.01), RVEDVI (Pearson r = 0.376; *p* = 0.001), RVESVI (Pearson r = 0.358 *p* = 0.002), RVEF (Pearson r = −0.282; *p* = 0.018) and NT pro-BNP (Pearson r = 0.392; *p* = 0.001), but not to CI, PVR, RVSVI and mPAP (Fig. 4).

### Comparison of native T1-values between IPAH, CTEPH and PAH-Ssc patients and control subjects

Native T1-values of the RV free wall, LV free wall, interventricular septum and the interventricular insertion regions were not significantly different between IPAH, CTEPH and PAH-SSc patients.

**Fig. 5:**
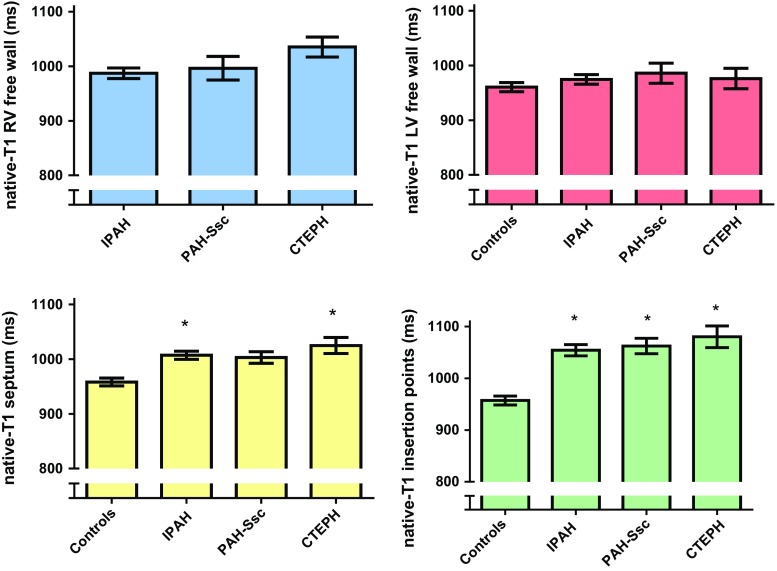
Comparison of native T1-values between IPAH, PAH-SSc and CTEPH patients and control subjects. Data is presented as mean and standard error of the mean. The Y-axis Native T1-values of the RV free wall (*blue*), LV free wall (*red*), interventricular septum (*yellow*) and interventricular insertion regions (*green*) were not significantly different between IPAH, PAH-SSc and CTEPH patients. Native T1-values of the LV free wall was not significantly different between control subjects and PH patients. Native T1-values of the interventricular septum was significantly higher in IPAH and CTEPH patients compared to control subjects. Native T1-values of the interventricular insertion regions were significantly increased in all PH categories compared to control subjects

Native T1-values of the LV free wall were not significantly different between control subjects and PH patients. Native T1-values of the interventricular septum was significantly higher in IPAH and CTEPH patients compared to control subjects. Native T1-values of the interventricular insertion regions were significantly higher in all PH categories compared to control subjects (Fig. 5).

## Discussion

This is the first study using the native T1-mapping technique to characterize the myocardium in precapillary PH patients. In PH patients, native T1-values of the interventricular insertion regions were significantly increased compared to the native T1-values of the RV free wall, LV free wall and interventricular septum. Furthermore, native T1-values of the interventricular insertion regions were related to disease severity.

### Increased native T1-values of the interventricular insertion regions

LGE studies in PH showed the late enhancement of the interventricular insertion regions and it has been suggested that this phenomenon most likely reflect locally increased focal fibrosis [2–5]. This suggestion was strengthened by McCann et al. [4] by finding increased focal fibrosis at the interventricular insertion regions in the histology of the myocardium of two PH patients. Bull et al. [12] linked the native T1-values to histological findings in patients with severe aortic stenosis and found a good correlation between native T1-values and the collagen volume fraction. Furthermore, a recent study investigating native T1-values in a chronic PH animal model also found increased native T1-values at the interventricular insertion regions. In this study, the PH group showed increased interstitial collagen at the interventricular insertion regions compared to the sham group [27]. Therefore, the increased native T1-values of the interventricular insertion regions most likely reflect locally increased focal fibrosis. However, McCann et al. [4] also described edema at the interventricular insertion regions, which also can contribute to an increase in native T1-values [17]. It is suggested that predilection for fibrosis to develop at the interventricular insertion regions is caused by mechanical stress due to the bowing of the interventricular septum into the LV [2] which is often seen in precapillary PH patients. However, late enhancement at the interventricular insertion regions is also described in patients with hypertrophic cardiomyopathy [3, 28] indicating that this phenomenon is not specific for a pressure overloaded RV.

Increased native T1-values of the interventricular insertion regions were moderately, but significantly related to disease severity. This is in line with previous LGE studies showing comparable correlations between the late enhancement of the interventricular insertion regions and RVEF, RV volumes and mPAP [2, 4, 5]. Furthermore, similar correlations were found between native T1-values of the interventricular insertion regions and measures of disease severity [27]. The correlations between native T1-values of the interventricular insertion regions and RV functional measures and NT-proBNP is probably related to the associated increase in RV wall tension. It has been shown that RV wall tension is associated with the delay in time to peak shortening of the RV, causing the leftward shift of the interventricular septum during late RV systole [29]. The right-to-leftward shifting of the interventricular septum probably increases the mechanical shear stress on the interventricular insertion regions.

In a recent study, native T1-values were assessed in the RV of healthy subjects and the authors found increased native T1-values in the RV free wall compared to the LV free wall and suggested that this finding could be due to a higher collagen content of the RV free wall [30]. In the PH patients in our study, native T1-values of the RV free wall were not significantly higher than those of the LV free wall and were in the same range as the native T1-values of the RV free wall in healthy subjects [30]. We could not assess the native T1-values of the RV wall in our control subjects because the wall was too thin. Kawel-Boehm et al. [30] could assess native T1-values of the RV free wall of healthy subjects because they performed native T1-mapping at the end-systolic phase.

Native T1-values of the LV free wall were not significantly different between PH patients and control subjects and were in the same range as native T1-values of the LV free wall conducted in a large cohort of healthy subjects of the same age [11]. This is in line with LGE studies showing no late enhancement in the myocardium of precapillary PH patients apart from the interventricular insertion regions [2–5].

### Native T1-values between IPAH, PAH-SSc and CTEPH patients

A recent study of Ntusi et al [18] found increased native T1-values of the total myocardium in no-PH systemic sclerosis patients compared to controls. We found no differences in native T1-values between IPAH, PAH-SSc and CTEPH patients. The presence of myocardial fibrosis found in these different forms of precapillary PH differ between studies. No differences in myocardial fibrosis of RV free wall tissue were found between controls, IPAH and PAH-SSc patients [21], while others found an increased amount of myocardial fibrosis in PAH patients compared to no-PH controls [22]. Ntusi et al. [18] included both limited cutaneous (lcSSc) and diffuse cutaneous systemic sclerosis (dcSSc) patients, while in our study we only included patients with lcSSc. It is known that cardiac involvement of systemic sclerosis is much higher in dcSSc patients compared to lcSSc patients [31], which can explain the differences in results.

## Limitations

We could not assess native T1-values of the total RV free wall, since in most patients the RV free wall was too thin to avoid partial volume effects. Only the inferior part of the RV free wall could be accurately assessed in all patients. Although we did not assess the total RV free wall, large variances in native T1-values between different regions of the RV free wall are not expected in a pressure overloaded RV.

Our study is a retrospective analysis and we included both treatment naïve and treated PH patients. From our data we cannot rule out any treatment effects on the measured native T1-values.

The control subjects were significantly younger compared to PH patients. It is known that age can influence native T1-values of the myocardium, with higher native T1-values found at a younger age [11]. Since native T1-values of the interventricular insertion regions were significantly increased in PH patients compared to control subjects, this only strengthens our findings.

The MOLLI technique as we applied is known to slightly underestimate native T1 values at higher heart rates [32]. The main conclusions in this study rely on regional differences, and are therefore not affected. Moreover, heart rates were not different between patients and controls. Another potential confounder is the effect of the T2 relaxation time: the absolute T1 values obtained with the MOLLI technique defer from their actual values in tissues with short T2 [33]. Whether or not T2-changes play a role in our study, is still to be explored. A technique such as SASHA [34] is more accurate, however, the values obtained with this SACHA technique show a larger variability and the MOLLI method performs more precisely [35].

## Conclusions

Native T1-values of the interventricular insertion regions are increased compared to native T1-values of the LV free wall, RV free wall and interventricular septum in patients with precapillary PH and are related to disease severity. Native-T1 values are not different between IPAH, PAH-SSc and CTEPH patients. Native T1-mapping can be an alternative for the characterization of the interventricular insertion regions without the use of contrast agents.
